# Probucol: revisiting as a multifaceted therapeutic agent in atherosclerosis

**DOI:** 10.3389/fphar.2025.1704983

**Published:** 2026-01-12

**Authors:** Sha Li, Hui-Hui Liu, Rui-Xia Xu, Jian-Jun Li

**Affiliations:** Cardiometabolic Center, State Key Laboratory of Cardiovascular Disease, Fuwai Hospital, National Center for Cardiovascular Diseases, Chinese Academy of Medical Sciences, Peking Union Medical College, Beijing, China

**Keywords:** probucol, cardiovascular disease, lipid metabolism, antioxidant drug, atherosclerosis

## Abstract

Atherosclerotic cardiovascular disease (ASCVD) is known to be driven by chronic inflammation and pro-atherogenic dyslipidemia and remains as one of the leading causes of mortality globally. Despite the success of current lipid-lowering therapies, a significant residual inflammatory risk persists, highlighting the need for treatments with broader mechanisms. Probucol is a lipid-modulating agent with uniquely potent antioxidant and anti-inflammatory properties that is re-emerging as a compelling multifunctional drug against atherosclerosis. This review critically examines the available evidence on probucol’s impacts on ASCVD, with special focus on its integrated effects on the cholesterol–inflammation pathway. We examine the pleiotropic mechanisms of this drug, including the inhibition of low-density-lipoprotein oxidation and reverse cholesterol transport potentiation, as well as appraise the clinical data demonstrating its ability to regress atherosclerotic plaques and reduce cardiovascular events. While acknowledging the controversies that have limited the use of probucol, such as its high-density-lipoprotein cholesterol-lowering effect and risk of QT prolongation, we provide an updated and balanced perspective on its risk–benefit profile. By compiling existing knowledge from mechanistic and clinical studies, this review argues for a reappraisal of probucol’s role and outlines future directions needed to establish its place in the modern management of atherosclerosis.

## 1 Introduction

Atherosclerotic cardiovascular disease (ASCVD) is a chronic inflammatory disorder that remains as one of the foremost threats to health globally. Although the advent of statins marked a turning point in lowering low-density-lipoprotein cholesterol (LDL-C), there remains a substantial residual risk of cardiovascular events that is largely driven by unresolved inflammation and oxidative stress. This therapeutic gap underscores the need for interventions with pleiotropic mechanisms that extend beyond lipid reduction.

Probucol is a molecule that was originally synthesized as a potent antioxidant but warrants a critical reappraisal in this modern context. It was approved in 1977 as a lipid-lowering agent but was soon marginalized by statins and concerns over its concurrent lowering of high-density-lipoprotein cholesterol (HDL-C). However, long-term clinical evidence, particularly from Japan, has consistently suggested its powerful anti-atherosclerotic efficacy. These benefits appear to be mediated primarily by its profound antioxidant and anti-inflammatory properties, making it an ideal candidate to target the residual risk unmet by current therapies. The present review therefore critically evaluates the available mechanistic and clinical evidence with the aim of redefining probucol’s role in the contemporary management of ASCVD.

## 2 Pharmacological role of probucol in the modulation of atherosclerosis

### 2.1 Regulating LDL: beyond receptor-dependent pathways

Probucol is a lipid-lowering drug that can lower circulating LDL-C by increasing the cellular uptake and degradation of LDL through both the LDL receptor (LDLR)-dependent and LDLR-independent pathways (Yamashita and Matsuzawa, 2009). Probucol is efficient in LDLR-deficient animal models and familial hypercholesterolemia (FH) patients as well as in reducing the synthesis of apoB, which is a major structural protein of LDL essential for recognizing LDLR that reduces nascent particle formation and promotes LDL clearance (Frikke-Schmidt et al., 2008). Although the complete mechanism of probucol has not been explained fully, its effect of lowering LDL-C via structural modification and metabolic modulation on LDL particles bears some evidence. Thus, probucol should have specific value from the view of a traditional cholesterol-lowering drug to patients who suffer from a residual risk of statin usage (Frikke-Schmidt et al., 2008).

### 2.2 Modulating HDL: beyond concentration metrics

To date, various strategies for increasing HDL-C have been found to not be positive. The cholesterol ester transfer protein (CETP) inhibitors like torcetrapib, dalcetrapib, and evacetrapib have not shown significant reductions in major cardiovascular events (CVEs) (Lincoff et al., 2017), although anacetrapib showed modest efficacy but was discontinued because of concerns regarding its tissue-retention half-life and suboptimal clinical effects (Bowman et al., 2017). These therapeutic inadequacies may result from CETP inhibition producing dysfunctional HDL with compromised atheroprotective functions (Krishna et al., 2017).

However, probucol’s mechanism of modulating HDL quantity and/or quality makes it a promising candidate for current management of ASCVD. It can suppress hepatic apoA-I and apoA-II synthesis, resulting in decreased HDL-C levels. Further, it can stimulate reverse cholesterol transport (RCT) by upregulating key regulators like scavenger receptor class B type I (SR-BI), adenosine triphosphate (ATP)-binding cassette (ABC) transporter A1 (ABCA1), and paraoxonase-1 (PON1) (Sasaki et al., 2019). Hepatic SR-BI expression has been shown to increase in humans and rabbits with enhanced selective uptake of cholesterol esters from HDL, while macrophage and hepatic ABCA1 expressions are shown to increase to augment cellular cholesterol efflux and PON1 enzymic activity, which reinforce the antioxidant functions of HDL (Zhong et al., 2011). Moreover, probucol simultaneously activates CETP-mediated neutral lipid transfer to promote cholesterol flux toward the hepatocytes. Thus, complex HDL-focused studies have shown a critical shift away from number toward HDL functionality by focusing specifically on its efflux capacity (Sacks and Jensen, 2018). A review of the functionality can redirect our focus on quantity assessment to the qualities of HDL-C, including elevated preβ-HDL subspecies and increased cholesterol efflux, rather than quantitative changes even with the observed drop in total serum HDL-C. The therapy-driven changes to the HDL profile in the probucol group have shown improved functions and thus present interesting opportunities for the modification of existing ASCVD care options (Inagaki et al., 2012).

### 2.3 RCT: enhancement and optimization

**FIGURE 1:**
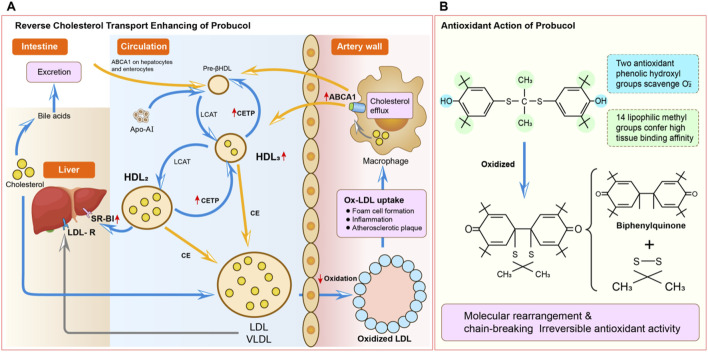
Proposed mechanisms for the anti-atherosclerotic actions of probucol. **(A)** Modulation of reverse cholesterol transport (RCT): The RCT pathway begins with the uptake of oxidized low-density lipoprotein (ox-LDL) by macrophages. The subsequent efflux of free cholesterol from these lipid-laden macrophages (foam cells) is actively mediated by the ATP-binding cassette transporter A1 (ABCA1). Pre-βHDL and lipid-poor HDL_3_ accept this cholesterol, maturing into larger and lipid-rich HDL_2_ particles. Cholesterol is then returned to the liver for excretion via two routes: a direct route wherein HDL_2_ binds to the hepatic scavenger receptor class B type I (SR-BI) and an indirect route in which cholesteryl ester transfer protein (CETP) facilitates the exchange of cholesterol esters from HDL_2_ for triglycerides in LDL. The resulting LDL is then taken up by the liver’s LDL receptors. Probucol enhances this process by notably increasing CETP-mediated cholesterol flux and excretion. **(B)** Intrinsic antioxidant activity: Probucol’s highly lipophilic nature enables it to integrate deeply with the lipid cores of LDL particles. Its bisphenol structure provides potent antioxidant capacity, acting as a scavenger of reactive oxygen species (ROS). In an oxidative environment, probucol is preferentially oxidized before the lipids or apolipoproteins of the LDL particles. This protective mechanism prevents the generation of pro-atherogenic ox-LDL and proceeds through an irreversible reaction, where probucol is first oxidized to a transient radical intermediate that then undergoes molecular rearrangement and C-S bond scission to form a benzoquinone metabolite. ABCA1, ATP-binding cassette transporter A1; CETP, cholesteryl ester transfer protein; HDL, high-density lipoprotein; LDL, low-density lipoprotein; LDL-R, low-density lipoprotein receptor; ox-LDL, oxidized low-density lipoprotein; RCT, reverse cholesterol transport; ROS, reactive oxygen species; SR-BI, scavenger receptor class B type I.

RCT is an important route for cholesterol clearance; here, HDL-apoA-I initiates the efflux of cholesterol from the foam cells by ABCA1/ABCG1, while lecithin cholesterol acyltransferase (LCAT) esterifies free cholesterol to cholesterol esters in the HDL particles and CETP mediates the exchange of these cholesterol esters between HDL and apoB-containing lipoproteins; finally, these are cleared through the hepatic LDLR-dependent uptake of lipoproteins or direct hepatic SR-BI-dependent internalization of HDL-C esters, facilitating biliary excretion (Smith, 2010). It has been proposed that RCT may be responsible for the lipid-lowering effects of probucol (Figure 1). In models of hypercholesterolemia, probucol was found to upregulate hepatic SR-BI expression, leading to accelerated direct HDL-C ester clearance along with stimulation of the LDLR-dependent pathways (Takiguchi et al., 2018). There is additional efflux of cholesterol from the macrophages to feces independently of SR-BI, which may be due to the increased fecal output of sterols derived from HDLs in murine models regardless of knockout at the SR-BI allele or wild type (Zhong et al., 2011). The oxidative metabolites of probucol like spiroquinone and diphenoquinone activate transcriptional upregulation of the ABCA1 transporters expressed on macrophages and hepatic cells with concomitant elevation of the plasma HDL-C levels, thus enhancing the system-wide RCT efficiency. Probucol decouples the efficacy of the RCT process independent of the circulating quantity of HDL-C, along with enhanced macrophage cholesterol clearance and hepatic processing, while reducing the total circulating level of HDL-C, thereby establishing it as a unique controller of the quality of cholesterol flux rather than that of lipoprotein abundance (Yakushiji et al., 2016).

### 2.4 Antioxidant/anti-inflammatory effects: multifunctional therapeutic basis

Better understanding of probucol has allowed examination of its effectiveness in terms of not only lipid modulation but also antioxidant and anti-inflammatory properties (Jackson et al., 1991). Such activities are a result of the unique bisphenolic structural motif where two phenolic hydroxyls are preferentially oxidized. Upon meeting oxygen free radicals, probucol donates electrons to form stable probucol radicals that then interrupt the lipid peroxidation chain reaction (Kaminnyi et al., 2007). Such unique antioxidant effects provide specific protection against lipoprotein oxidation. Moreover, one of the crucial benefits of probucol treatment is inhibition of the formation of oxidized LDL (ox-LDL) that plays a vital role in the pathogenesis of atherosclerosis. Probucol inhibits cell-mediated and transition-metal-catalyzed LDL oxidation (Kita et al., 1992), while also reducing monocyte adhesion, macrophage phagocytic activity, and foam cell transformation. It was shown to avoid atherosclerosis and myocardial injury in Watanabe heritable hyperlipidemic (WHHL) rabbits without changing the levels of alpha tocopherol and ubiquinone 10, while also protecting endothelial functions by downregulating the expression of vascular cell adhesion molecule-1 (VCAM-1) (Zapolska-Downar et al., 2001). Then, it induces heme oxygenase-1 (HO-1) to inhibit the proliferation of vascular smooth muscle cells (VSMCs), modulate the redox status, and decrease the endogenous nitric oxide (NO) synthase inhibitors to enhance vasomotor functions. Additionally, it enhances PON1 activity to boost the ability of HDL to prevent LDL peroxidation while facilitating HDL functionalization as well as shows synergism in reducing atherogenesis with cilostazol and atorvastatin combination therapy in a moderate hypercholesterolemic model (Wang et al., 2015). Probucol also impairs the inflammatory differentiation of monocytes and diminishes generation of reactive oxygen species (ROS) by repressing L-homocysteine-induced nicotinamide adenine dinucleotide phosphate (NADPH) oxidase activation (Zhang et al., 2018).

**FIGURE 2:**
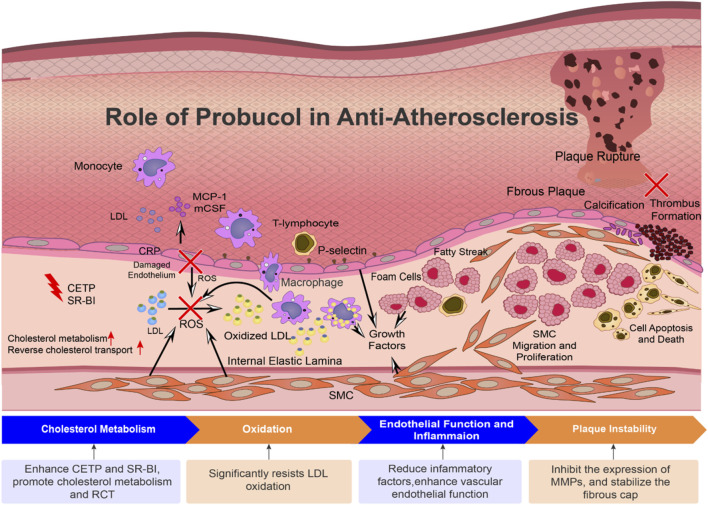
Role of probucol in anti-atherosclerosis. Probucol exerts its therapeutic actions through multiple synergistic mechanisms. (1) It enhances RCT by increasing the activities of CETP and SR-BI. (2) Its potent antioxidant properties inhibit the oxidation of LDL, thereby reducing foam cell formation and decreasing atherosclerotic plaque volume. (3) Probucol mitigates vascular inflammation, as indicated by the reduced levels of hs-CRP and other inflammatory mediators, which in turn improves endothelial function and promotes endothelial regeneration. (4) These actions collectively contribute to the stabilization of vulnerable plaques by inhibiting matrix metalloproteinase (MMP) expression, reinforcing the fibrous cap, and suppressing smooth muscle cell migration. This plaque-stabilizing effect is the primary mechanism responsible for reducing major adverse cardiovascular and cerebrovascular events. CETP, cholesteryl ester transfer protein; CRP, C-reactive protein; LDL, low-density lipoprotein; MCP-1, monocyte chemoattractant protein-1; M-CSF, macrophage colony-stimulating factor; MMPs, matrix metalloproteinases; ox-LDL, oxidized low-density lipoprotein; RCT, reverse cholesterol transport; ROS, reactive oxygen species; SMC, smooth muscle cell; SR-BI, scavenger receptor class B type I.

These experimental results show that probucol is more conducive to preventing atherosclerosis given its combined lipid-dependent and non-lipid-dependent properties (Figure 2). *In vivo* preclinical studies have shown that compared with statin treatment, probucol could lower aortic lesions more significantly and increase monocyte adhesion (Niimi et al., 2013); it can also protect VSMCs from apoptosis, increase the collagen thickness of the arterial fibrous caps, and stabilize soft plaques. Furthermore, the serum inflammatory factors and matrix metalloproteinases (MMPs) were found to be lowered, and the toll-like receptor expressions were reduced. When combined with atorvastatin, probucol showed synergistic anti-atherosclerotic effects in apoE-knockdown mice (Guo et al., 2019). Probucol can also improve myocardial functions, extended the life span in SR-BI, modulate miR-497 expression, and regulate MMP-9 expression (Wang et al., 2016). In diabetic LDLR^−/−^ mice, probucol reduced atherosclerosis by lowering cholesterol levels and blocking the maturation of dendritic cells (Li et al., 2021). Probucol was shown to protect against endothelial precursor cell injury induced by ox-LDL, while inhibiting the associated inflammation and elastin degradation (Chen et al., 2018). In addition, probucol’s enhancement of CETP activity against arteriosclerosis may provide new drug targets (Schwartz et al., 2012). Given its superior preclinical performance and pleiotropic effects on lipid regulation as well as antioxidant and anti-inflammatory effects, probucol has broad prospects in atherothrombotic therapy.

### 2.5 Probucol in the modern therapeutic landscape: a comparative perspective

The re-evaluation of probucol necessitates a critical appraisal of its position relative to established and emerging therapies against atherosclerosis. While the preceding sections have detailed the standalone pharmacological actions of probucol, its true potential can only be realized through a comparative lens. To this end, we provide a systematic comparison of probucol’s key pharmacological and clinical features against those of statins (Yang et al., 2023), PCSK9 inhibitors (Nicholls, 2023), CETP inhibitors (Nicholls and Nelson, 2022), and targeted anti-inflammatory agents (Wautier and Wautier, 2023) (Table 1). This comparative framework is essential for delineating the unique therapeutic niche of probucol.

**TABLE 1:** Critical comparison of probucol with key lipid-lowering and anti-inflammatory agents.

Feature	Probucol	Statin	PCSK9 inhibitor (PCSK9i)	Cholesteryl ester transfer protein (CETP) inhibitor	Anti-inflammatory agent
Primary mechanism of action	Lipid lowering: enhances LDL clearance via LDL-receptor-independent pathways and promotes reverse cholesterol transport (RCT) Anti-inflammatory/antioxidant: as a potent lipophilic antioxidant, it integrates into LDL particles, directly inhibiting their oxidation	Lipid lowering: inhibits HMG-CoA reductase to upregulate hepatic LDL receptor expression and increase LDL clearance Anti-inflammatory: exerts pleiotropic anti-inflammatory effects via complex mechanisms	Lipid lowering: monoclonal antibodies bind to and inhibit PCSK9, preventing LDL receptor degradation and substantially increasing the density of surface LDL receptors on hepatocytes	Lipid lowering: inhibits CETP to prevent transfer of cholesteryl esters from HDL to ApoB-containing lipoproteins, leading to a significant increase in HDL-C level	Anti-inflammatory: directly targets key inflammatory pathways, such as IL-1β (e.g., canakinumab) or inflammasome activation (e.g., colchicine), through a mechanism independent of lipid modulation
Impact on LDL-C	Moderate reduction	Potent reduction	Highly potent reduction (50%–60% further reduction on top of statin therapy)	Minimal reduction or no effect	No direct effect
Impact on HDL-C	Marked reduction	Mild elevation	Mild elevation	Marked elevation	No direct effect
Anti-inflammatory/antioxidant effects	Key feature: possesses direct and potent intrinsic antioxidant activity as a core mechanism to significantly inhibit ox-LDL formation	Advantage: established anti-inflammatory effects (e.g., reduces hs-CRP), considered a key component of the pleiotropic actions	Indirect effects, likely secondary to profound LDL-C lowering and subsequent improvements in endothelial function	Clinical benefits are unproven; failed to demonstrate a clear anti-inflammatory advantage	Core advantage: primary mechanism of action addresses and reduces residual inflammatory risk beyond statin therapy
Evidence for major clinical endpoints	Some trials show slowed progression of carotid intima-media thickness (FAST) and reduced post-PCI restenosis. Strong secondary prevention data in specific populations like FH (POSITIVE). A large trial (PROSPECTIVE) showed a trend toward reduced cardiovascular events	Cornerstone therapy: overwhelming evidence for significant reduction of MACCEs in both primary and secondary prevention settings	Robust evidence: conclusively shown to further reduce MACCEs on a background of statin therapy in high-risk patients (FOURIER, ODYSSEY OUTCOMES)	Largely unsuccessful (e.g., torcetrapib, dalcetrapib, and evacetrapib); failed to translate HDL-C elevation into clinical endpoint benefit	Positive evidence: proven to reduce MACCEs in select high-risk patient populations with residual inflammatory risk (CANTOS, COLCOT, and LODOCO2)
Distinct advantages	1. Potent and direct antioxidant activity 2. Improves HDL function (enhances RCT) rather than merely increasing HDL-C quantity 3. Leads to regression of xanthomas in patients with FH 4. Possesses an LDL-receptor-independent mechanism for lipid lowering	1. Oral administration and low cost (generics available) 2. Vast body of clinical evidence and long-term safety data 3. Cornerstone of lipid-lowering therapy owing to proven efficacy	1. Most potent LDL-C reduction available 2. Infrequent subcutaneous administration (biweekly or monthly), potentially improving adherence 3. Provides a powerful option for statin-intolerant patients or those with inadequate response	1. Most potent agent for raising HDL-C levels (although this has not translated to clinical benefit)	1. Specifically targets residual inflammatory risk, offering a new dimension for ASCVD secondary prevention beyond lipid lowering 2. Complementary mechanism of action to lipid-lowering therapies
Potential drawbacks and side effects	1. Reduces HDL-C level, which is counterintuitive to conventional lipid theory 2. Potential for QT interval prolongation, raising concerns of arrhythmia (although fatal arrhythmias did not increase in major trials) 3. Gastrointestinal side effects (e.g., diarrhea)	1. Muscle-related side effects (myalgia and myopathy) 2. Small increased risk of new-onset diabetes 3. Elevation of liver enzymes	1. High cost 2. Injection-site reactions 3. Requires administration by injection	1. Lack of clinical endpoint benefit; some agents increased risk (e.g., torcetrapib) 2. May produce dysfunctional HDL particles	1. Increased risk of infection (especially canakinumab) 2. Gastrointestinal intolerance (colchicine) 3. Requires careful patient selection (e.g., elevated hs-CRP) for benefit

Abbreviations: ASCVD, atherosclerotic cardiovascular disease; CETP, cholesteryl ester transfer protein; FH, familial hypercholesterolemia; HDL-C, high-density-lipoprotein cholesterol; hs-CRP, high-sensitivity C-reactive protein; IL-1β, interleukin-1 beta; LDL-C, low-density-lipoprotein cholesterol; MACCE, major adverse cardiovascular and cerebrovascular events; ox-LDL, oxidized low-density lipoprotein; PCI, percutaneous coronary intervention; PCSK9, proprotein convertase subtilisin/kexin type 9; RCT, reverse cholesterol transport.

As illustrated in Table 1, probucol does not compete with the frontline efficacies of statins or PCSK9 inhibitors in terms of only the LDL-C-lowering potential. Instead, its therapeutic rationale is rooted in a distinct and multifaceted action mechanism that addresses different pathophysiological pathways. Foremost among these actions is its profound and direct antioxidant activity as a feature not intrinsic to other lipid-modifying agents that prevents the formation of pro-atherogenic oxidized LDL, which is a key initiating step in atherogenesis.

Probucol also challenges the conventional “higher-is-better” paradigm for HDL-C. Although it paradoxically reduces HDL-C levels, a finding that contributed to its initial marginalization, emerging evidence suggests that it fundamentally improves HDL functionality. By activating CETP and enhancing RCT, probucol promotes the efflux of cholesterol from peripheral tissues for excretion. This focus on cholesterol efflux and particle quality is in stark contrast with CETP inhibitors, which potently elevate HDL-C quantity but fail to translate to consistent clinical benefits, highlighting that particle function is more critical than concentration.

This unique pharmacological profile with its inherent strengths (direct antioxidant action, RCT enhancement) and acknowledged limitations (modest LDL-C reduction, HDL-C lowering, QT prolongation) provides an essential context for interpreting the clinical evidence accumulated over decades. The following sections explore how these mechanistic attributes have been translated into clinical outcomes across a spectrum of cardiovascular and metabolic diseases.

## 3 Probucol in cardiovascular disease management

### 3.1 Probucol in the management of atherosclerosis

Rethinking the role of probucol in current anti-atherosclerotic therapy would be a reasonable choice. The probucol quantitative regression Swedish trial (PQRST) was initially designed to quantify the effects of probucol on femoral atherosclerosis in subjects with hypercholesterolemia via angiographic methods. The primary endpoint here was the change in femoral artery lumen volume as quantified by quantitative angiography. However, in reality, probucol-treated individuals showed 12% lower LDL-C and 24% lower HDL-C levels compared to controls but no remarkable differences concerning lumen volume (Walldius et al., 1994). Later, the Fukuoka atherosclerosis trial (FAST) illustrated that probucol can decrease carotid intima-media thickness (CIMT) progression and reduce the incidence of CVEs; here, asymptomatic hypercholesterolemic subjects were randomized into one of three groups (probucol 500 mg/d, pravastatin 10 mg/d, or diet modification only) over a period of 2 years. Subjects who were allotted to probucol and statin treatments experienced a 13.9% reduction in IMT levels (*p* < 0.01) vs. a 23.2% increase in control subjects (*p* < 0.05). Notably, the cardiac event rate in the probucol group was markedly lower than in the control group at 2.4% vs. 13.6% (Sawayama et al., 2002). These observations suggest that probucol may be effective in the prevention of ASCVD.

Another remarkable study reported is the probucol trial for secondary prevention of atherosclerotic events (PROSPECTIVE) in patients with prior coronary heart disease (CHD), which was a multicenter, randomized, prospective trial performed in Japan on 876 subjects having CHD and dyslipidemia characterized by an LDL/HDL-C value >140 mg/dL who have never received lipid-lowering therapy (Yamashita et al., 2021). For almost 3 years, all subjects randomly chosen for the control arm were given standard lipid-lowering drugs while the probucol group received an extra benefit of 500 mg/d of probucol in addition to standard lipid-lowering drugs. Importantly, although probucol reduced the HDL-C level, it actually decreased these events instead of elevating them. Thus, it appears that probucol could alter our understanding of cardiovascular health.

Another pioneering multinational, open-label, blinded-endpoint study evaluated the long-term effects of probucol alone and in combination with cilostazol and statins on the mean CIMT (Kang et al., 2021). Here, patients with CHD and hyperlipidemia were randomized to three groups under statin monotherapy, statins and probucol, or a combination of all three therapies over 3 years. The primary endpoint was the change in CIMT at 3 years, while the secondary outcomes included biomarkers, major adverse cardiovascular and cerebrovascular events (MACCEs), and safety. In the first year, the combination group showed significantly greater decrease in CIMT than the control group, while there were no significant differences in CIMT changes between the groups by the third year (control: −0.12 ± 0.36 mm vs. probucol: −0.11 ± 0.32 mm, combination:−0.16 ± 0.38 mm). There were no significant differences in the event rates (numerically higher in the control arm) shown below (control: 10.8% vs. probucol: 4.4%, combination: 6.9%) (*p* = 0.35), and both probucol and cilostazol exhibited good tolerances without serious drug-related adverse reactions. A comprehensive analysis indicated a trend toward reduction of CVEs for probucol, with an adjusted hazard ratio (HR) of 0.67 (95% confidence interval (CI): 0.44–1.03) (Arai et al., 2022). Although there were no significant differences in CIMT between the treatment groups, patients with HDL-C levels (≥6.25 mg/dL) modified by probucol showed lower risk of events.

Thus, the anti-atherosclerosis effects of probucol rely mostly on its antioxidant properties and facilitator role in RCT (Li et al., 2011). Hence, it demonstrates impressive results in clinical applications against atherosclerosis and in the secondary prevention of ASCVD. The novel action mechanism of probucol thus holds importance when added in current therapeutic regimens.

### 3.2 Probucol in percutaneous coronary intervention

Probucol has demonstrated efficacy in preventing restenosis and reducing the occurrence of MACCEs after percutaneous coronary intervention (PCI) procedures, thereby improving long-term prognosis. In a double-blind randomized trial, patients who received probucol treatment for 4 weeks before and 6 months after PCIs had lower luminal diameters and segment-wise restenosis rates compared to those who received multivitamins and placebo (Tardif et al., 1997; Côté et al., 1999). A meta-analysis showed that probucol significantly decreased the incidence of restenosis after PCI (*p* = 0.0007) and vascular restenosis (*p* < 0.00001). Meanwhile, the incidence of MACCEs was significantly lower in the probucol group than control group (relative risk (RR): 0.69; *p* = 0.01) (Liu et al., 2015). Similar results were reported for the probucol angioplasty restenosis trial (PART) (Yokoi et al., 1997). Researchers also investigated the influence of probucol treatment on the long-term survival rates of CHD patients after complete revascularization, including PCI or/and bypass (n = 1694); the analysis results showed that the all-cause mortality risk of the probucol group was significantly lower than that of the control group that did not receive probucol treatment (HR: 0.45; *p* = 0.002) (Kasai et al., 2012). The intracoronary stenting and angiographic results of the test efficacies of the sirolimus-, probucol-, and zotarolimus-eluting stents (ISAR-TEST 5) trial with 10-year follow-up demonstrated that probucol-eluting stents were not superior for long-term outcomes and stent thrombosis rates compared to the zotarolimus-eluting stents (Koch et al., 2021; Kufner et al., 2020). Another study showed that the probucol-coated non-polymeric ultrathin-strut sirolimus-eluting stent showed significantly better early strut coverage at 3 months (Otaegui et al., 2022). Interestingly, probucol could prevent kidney damage by iodinated contrast media used in interventional therapy; it was shown to reduce oxidative stress in the kidneys, accelerate renal recovery, and protect the kidneys from contrast-induced acute kidney injury (CIAKI) after primary or emergency angioplasty (Endo et al., 2013). The risk of CIAKI was significantly lower after the use of a hydrating solution and probucol than hydration alone, and the incidence rate of contrast-induced nephropathy (CIN) was also reduced between groups from 10.9% to 4.0% by probucol with hydration after PCI (Fu et al., 2018). These positive results have also been confirmed by other studies (Xin et al., 2019).

### 3.3 Probucol in the management of ischemic stroke

Probucol has been shown to have anti-neuroinflammatory properties and protective effects against cerebral ischemia. Among the three main preventive measures for ischemic stroke, namely, antiplatelet agents, blood pressure control, and lipid-lowering therapy, the latter has been firmly established and holds a strong position. For those at high risk where traditional treatments may be insufficient or intolerable, probucol could be a valuable alternative (Lang et al., 2023). Through its anti-neuroinflammatory features, probucol was shown to inhibit microglia, reduce the infarction volume, and improve cerebral ischemic damage in normal and hyperlipidemic mice (Jung et al., 2016). A double-blind, placebo-controlled, randomized clinical trial performed across Asia tested cilostazol and aspirin with and without probucol in ischemic stroke patients at high risk of developing intracranial hemorrhage (Kim et al., 2018); here, the researchers considered four treatment groups, where patients were administered daily doses of cilostazol, aspirin, cilostazol + probucol, and aspirin + probucol. The primary endpoints were incidence of stroke, myocardial infarction (MI), vascular death, and hemorrhagic stroke. Patients treated with probucol showed significantly lower event rates than the untreated groups (HR: 0.69; 95% CI: 0.50–0.97; *p* = 0.0316), suggesting that the addition of probucol could reduce event risk in patients with ischemic stroke. Furthermore, the combination of probucol and cilostazol might enhance vascular functions in patients with silent lacunar infarcts and mild hypercholesterolemia because these two medicines have beneficial effects on endothelial functions and lipid profile (Takechi et al., 2014). There was also no evidence that probucol has positive effects on cognition post-stroke, as measured by the Montreal Cognitive Assessment (MoCA) tool (Lim et al., 2021). Probucol’s cholesterol-lowering and antioxidant properties thus make it a promising option for managing both bleeding and ischemic risks in patients with cerebral infarction (Lang et al., 2024).

### 3.4 Probucol in the treatment of FH

**FIGURE 3:**
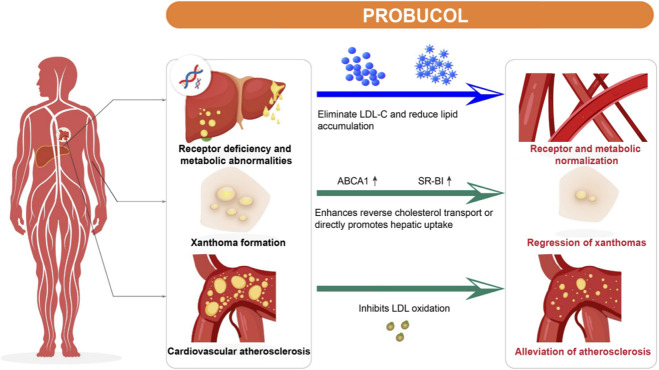
Role of probucol in familial hypercholesterolemia. Mutations in the LDL receptor gene in patients with familial hypercholesterolemia result in the liver being unable to metabolize LDL properly, leading to the formation of yellow nodules on the skin surface. Probucol clears LDL through non-receptor pathways to reduce lipid accumulation in the skin and tendons. It also promotes RCT by upregulating ABCA1 and SR-BI to directly promote cholesterol uptake by the liver. In addition, probucol inhibits LDL oxidation and reduces oxidized LDL levels. These combined effects lead to regression of xanthomas and alleviation of cardiovascular atherosclerosis, emphasizing its therapeutic potential in familial hypercholesterolemia. LDL, low-density lipoprotein; ABCA1, ATP-binding cassette transporter A1; SR-BI, scavenger receptor class B type I.

FH often presents with high serum LDL-C levels and causes early-age onset of ASCVD. Xanthomas are another important clinical symptom used to diagnose FH. The regression of xanthomas indicates the efficiency of lipid-lowering therapy (Kastelein et al., 2020). Probucol exhibits considerable effects on the treatment of FH and regression of xanthomas (Figure 3). A cohort study performed in Japanese patients with heterozygous FH with a follow-up period of up to 20 years indicated that long-term probucol use significantly prevented secondary CVEs (HR: 0.13; 95% CI: 0.05–0.34) (Yamas et al., 2008).

### 3.5 Bridging the bench and bedside: a consolidated view of the evidence

The above systematic review of the preclinical evidence (Section 2) and data from human clinical trials (Section 3.1, Section 3.2, Section 3.3, Section 3.4) reveal a comprehensive picture of the role of probucol in atherosclerosis. To facilitate a direct comparison, we compile these findings in Supplementary Table S1 by juxtaposing the potent effects observed at the bench with the outcomes documented at the bedside.

This comparison reveals a narrative of remarkable consistency in several key areas. There is strong concordance between the preclinical and clinical data regarding probucol’s ability to inhibit LDL oxidation, exert anti-inflammatory effects, and favorably impact direct measures of vascular health. This is best exemplified by its robust and repeatedly observed successes in reducing surrogate markers like CIMT and post-procedural restenosis.

However, the comparison highlights the central paradox of probucol, namely, the challenge of translating these potent vasculoprotective actions and surrogate marker improvements into definite reductions of the “hard” clinical endpoints (i.e., MACCEs) in some pivotal cardiovascular outcome trials. This apparent disconnect does not necessarily negate the biological efficacy of the drug but rather underscores the complexity of demonstrable event reduction while raising critical questions about historical trial designs and patient populations.

In summary, the weight of evidence confirms probucol as an agent with consistent and direct anti-atherosclerotic activities. While its ultimate impact on MACCEs in the broad populations remains a topic of scientific discussion, the proven benefits to the pathological processes underlying atherosclerosis provide a compelling rationale for its continued consideration in cardiovascular therapy.

## 4 Probucol in other diseases

Beyond its primary role in atherosclerosis, the potent antioxidant and pleiotropic properties of probucol have prompted investigations into its efficacy across a spectrum of other metabolic and inflammatory diseases. While these findings are secondary to the main focus of this review, they underscore the unique pharmacological profile of the compound. The key evidence from these studies is consolidated in Table 2, and a comprehensive discussion is available in the Supplementary Material.

**TABLE 2:** Summary of probucol’s pleiotropic effects in related metabolic and vascular conditions.

Therapeutic area/Disease	Key pharmacological mechanisms implicated	Summary of key preclinical and clinical evidence	Potential clinical relevance and implications
Type 2 diabetes mellitus (T2DM) and complications	Potent systemic antioxidant stress reduction Amelioration of endothelial dysfunction and enhancement of nitric oxide (NO) bioavailability Anti-inflammatory action to reduce pro-inflammatory cytokines (e.g., TNF-α and IL-6)	Clinical: demonstrated reduction in urinary albumin excretion, indicating a renoprotective effect in diabetic nephropathy Some studies report modest improvements in glycemic control and insulin sensitivity Preclinical: protects pancreatic β-cells from oxidative damage and apoptosis	Offers a potential strategy to mitigate diabetic micro- and macro-vascular complications (nephropathy, retinopathy, neuropathy), which share a common pathophysiology with ASCVD. Acts on the root causes of diabetic vascular damage and not just symptoms
Non-alcoholic fatty liver disease (NAFLD)/steatohepatitis (NASH)	Intense hepatic antioxidant effect to counteract lipid peroxidation Inhibition of pro-inflammatory cascades (e.g., NF-κB pathway) within the liver Modulation of hepatic lipid metabolism and VLDL export	Preclinical: consistently shows attenuation of steatosis, lobular inflammation, hepatocyte ballooning, and fibrosis in animal models of NASH Significantly reduces serum transaminases (ALT and AST) and hepatic markers of oxidative stress	Represents a strong therapeutic candidate for NASH, an independent risk factor for ASCVD, by targeting the “second hit” (inflammation and oxidative stress) of disease progression Potential for synergistic effects on both liver and cardiovascular health
Familial hypercholesterolemia (FH)	Promotion of non-traditional reverse cholesterol transport pathways (independent of ABCA1) Enhanced clearance of oxidized or otherwise modified LDL particles Potent LDL-C lowering (although less than statins)	Clinical: unique and proven efficacy in inducing significant regression of cutaneous and tendon xanthomas, often to a degree greater than that predicted by plasma LDL-C reduction alone Used as an adjunct therapy for managing extravascular cholesterol deposition	Highlights its distinct ability to mobilize cholesterol from tissue deposits, a feature not prominent with statins A valuable adjunct therapy in FH, especially for addressing the physical manifestations of cholesterol overload
Post-angioplasty restenosis	Antiproliferative effects on vascular smooth muscle cells (VSMCs) Suppression of neointimal hyperplasia following vascular injury Potent local anti-inflammatory and antioxidant actions at the site of intervention	Clinical: landmark clinical trials have demonstrated significant reductions in angiographic restenosis rates and the need for repeat revascularization following percutaneous coronary intervention (PCI) Benefit observed in both bare-metal and drug-eluting stent eras	Confirms its direct vasculoprotective role beyond systemic lipid lowering Suggests a niche application in interventional cardiology to improve long-term patency of treated vessels, addressing a key procedural complication
Neurodegenerative diseases (e.g., Alzheimer’s)	Neuroprotection via potent antioxidant activity within the central nervous system (CNS) High lipophilicity potentially allows it to cross the blood–brain barrier Reduction of lipid-peroxidation-mediated neuronal damage	Preclinical and speculative: animal models of Alzheimer’s disease and other neurodegenerative conditions suggest probucol can mitigate neuronal damage and preserve cognitive function Evidence remains preliminary and requires clinical validation	A speculative but mechanistically plausible area for future research, leveraging its antioxidant power to combat neuroinflammation and oxidative stress, which are the pathogenic pillars of these diseases Represents a frontier for repurposing probucol beyond cardiovascular medicine

For a detailed chapterwise discussions of the evidence summarized above, please refer to the Supplementary Material provided with this article.

## 5 Current recognition of probucol safety

The clinical utility of any therapeutic agent requires a comprehensive and balanced understanding of its safety profile. For probucol, this is characterized by the high incidence of manageable gastrointestinal side effects and at least one mechanistically understood cardiovascular concern, namely, prolongation of the QT interval.

### 5.1 General adverse reactions

The most frequently reported adverse effects of probucol are gastrointestinal in nature, including diarrhea, flatulence, abdominal pain, nausea, and vomiting (Kim et al., 2018). These symptoms are generally mild to moderately severe and often subside with continued therapy. A large Chinese study involving 561 patients reported an overall adverse effect rate of 3.74%, with gastrointestinal complaints being the most common; the authors speculated that this could be related to probucol’s lipophilicity and accumulation in the adipose tissues or its effects on HDL metabolism. Notably, the study showed no direct cardiovascular side effects (Tsui et al., 2023). Other less frequently reported effects include eosinophilia, dizziness, and headaches, which are broadly similar in character to those seen with other lipid-modifying agents.

### 5.2 QT interval prolongation: a critical risk–benefit assessment

The most significant safety consideration for probucol and a primary factor in its complex clinical history is its potential for prolongation of the QT interval. This issue warrants a thorough and critical evaluation by integrating mechanistic data with extensive evidence from clinical trials.

The concern for arrhythmia originated from early preclinical studies in the 1970s that showed that probucol could sensitize the canine myocardium to adrenaline-induced ventricular fibrillation (Marshall and Lewis, 1973). Modern molecular investigations have identified the mechanism underlying these observations: probucol can reduce the expression and function of the human ether-a-go-go-related gene (hERG) potassium channel (Shi et al., 2015). The hERG channel is critical for cardiac repolarization, and its inhibition is a well-established cause of drug-induced long-QT syndrome, which create an electrophysiological predisposition to arrhythmias. While some experimental compounds like matrine have been shown to counteract this effect *in vitro* (Shi et al., 2020), hERG inhibition remains the key pharmacodynamic basis for probucol’s pro-arrhythmic potential.

Consistent with its mechanism, clinical trials have confirmed that probucol therapy leads to a statistically significant prolongation of the QT interval compared to placebo (Tardif et al., 2003). An earlier report also highlighted that women may be more susceptible to probucol-associated tachycardia and long-QT syndrome (Reinoehl et al., 1996). However, a critical examination of the body of evidence reveals a crucial paradox: this electrocardiographic effect has not translated into increased rates of fatal ventricular arrhythmias in major clinical trials.

For instance, one placebo-controlled study noted that while there was increased incidence of QT prolongation, the absolute QT intervals of all patients remained within the normal range (Tardif et al., 2003). More importantly, a consistent finding across multiple large-scale secondary prevention trials, including prospective (Yamashita et al., 2021), positive (Yamas et al., 2008), Picasso (Kim et al., 2018), impact (Arai et al., 2022), and long-term follow-up (Kasai et al., 2012) studies, is that the observed QT prolongation was not accompanied by significant increases in the incidence of serious ventricular arrhythmias or sudden cardiac death.

This disconnect between the surrogate marker (QTc interval) and hard clinical outcomes necessitates a nuanced risk–benefit discussion. While the arrhythmogenic risk is not zero, the data from thousands of patients suggest that in monitored settings and in appropriately selected populations, the risk is low. The profound anti-atherosclerotic benefits of probucol may therefore outweigh its arrhythmogenic potential for many patients with high residual inflammatory risk.

In conclusion, while probucol’s potential to prolong the QT interval is a valid and mechanistically grounded concern, the extensive body of clinical evidence from major trials indicates that this does not lead to an overt increase in fatal arrhythmias. Therefore, the risk appears to be manageable with appropriate patient selection and vigilant clinical monitoring, allowing the unique therapeutic benefits of probucol to be leveraged in the right clinical context.

## 6 Conclusion and perspectives

### 6.1 Concluding summary: an established drug at a new crossroads

The extensive body of evidence reviewed herein reveals probucol as a therapeutic agent with a unique and multifaceted mechanism of action (Table 3). Unlike other lipid-modifying drugs, its clinical efficacy appears to be driven primarily by potent and direct antioxidant activity as well as a paradoxical but beneficial remodeling of HDL functions, which collectively inhibit the key pathological steps in atherogenesis. Although the clinical history of probucol has been marked by controversy, namely, its HDL-C-lowering effects and risk of QT prolongation, data from numerous trials have validated its ability to regress atherosclerotic plaques, prevent post-PCI restenosis, and reduce CVEs in specific populations. Probucol now stands at a critical crossroads: despite a strong mechanistic rationale and favorable data on the surrogate endpoints, its true potential in the contemporary management of ASCVD remains undefined owing to the lack of a definitive and large-scale outcomes trial.

**TABLE 3:** Notable clinical trials of probucol in various diseases over the past decades.

Research field	Trial	Country	Year of report	Participants characteristic	Participant	Intervention method	Follow-up period	Main result
Protective medication after intervention	MVP (Tardif et al., 1997)	Canada	1997	Patients scheduled for elective PCI with more than one *de novo* lesion (>50% luminal narrowing)	317	Probucol 500 mg twice daily, 4 weeks before, 6 months after vs. placebo, multivitamins (30,000 i.u. of b-carotene, 500 mg of vitamin C, and 700 i.u. of vitamin E), or both probucol and multivitamins	6 months	Restenosis rates per segment at 6 months: 20.7% in probucol arm (*p* = 0.003 vs. no probucol), 28.9% in combined (ns), 40.3% in multivitamins vs. 38.9% in control
Hyperlipidemia and cardiovascular disease	FAST (Sawayama et al., 2002)	Japan	2002	Patients with asymptomatic hypercholesterolemia	246	Probucol (500 mg/d), pravastatin (10 mg/d) or control (diet alone)	2 years	Probucol and pravastatin groups showed significant reductions in IMT (*p* < 0.01). Significantly lower incidence of cardiac events in probucol group than control group (*p* = 0.0136)
Hyperlipidemia and cardiovascular disease	PAB (Gallino et al., 2004)	Switzerland	2004	Patients with intermittent claudication after percutaneous transluminal angioplasty	335	Probucol 1000 mg daily, placebo, EVBT, and EVBT + probucol	6 months	Restenosis rate: 17% in EVBT, 35% in patients without EVBT (*p* < 0.001); 23% in probucol arm vs. 30% in placebo
Hyperlipidemia and cardiovascular disease	P + S (Tagawa et al., 2004)	Japan	2004	Patients with or without CHD	38	Probucol (500 mg/d, n = 9) or placebo (n = 9) treatment in patients with CHD	3 months	After probucol therapy, FMD was significantly improved in the CHD group (*p* < 0.05)
Hyperlipidemia and cardiovascular disease	Positive (Yamas et al., 2008)	Japan	2008	Patients with heterozygous FH	410	Lipid-lowering therapy with and without probucol (500–1000 mg/d)	Up to 20 years	Probucol lowered the risk (HR: 0.13; 95% CI: 0.050–0.34) in secondary prevention (n = 74) (*p* < 0.001)
Hyperlipidemia and cardiovascular disease	Not specified (Kasai et al., 2012)	Japan	2012	Patients who consecutively underwent complete revascularization	1,694	Lipid-lowering therapy with and without probucol (500–1000 mg/d)	10.2 ± 3.2 years	Risk of all-cause mortality was lower in the probucol group than the non-probucol group (*p* = 0.002)
Hyperlipidemia and cardiovascular disease	Picasso (Kim et al., 2018)	Korea	2018	Patients with ischemic stroke with a high risk of cerebral hemorrhage	1,512	A 2 × 2 factorial design: cilostazol versus aspirin; probucol versus no probucol	7 months	Addition of probucol to aspirin or cilostazol could be beneficial for reducing the incidence of cardiovascular events (*p* = 0.0316)
Hyperlipidemia and cardiovascular disease	Prospective (Yamashita et al., 2021)	Japan	2020	Patients with CHD who have received conventional lipid-lowering therapy	876	Lipid-lowering agents were administered during the study period in the control group (n = 438), and probucol 500 mg/d was added to lipid-lowering therapy in the probucol group (n = 438)	More than 3 years	Incidence of the primary endpoints showed a trend to be lower in the probucol group than the control group despite reduced HDL-C without serious adverse events
Hyperlipidemia and cardiovascular disease	Impact (Arai et al., 2022)	Korea	2021	Hypercholesterolemic patients with CHD	281	Control with statin alone; probucol group with statin and probucol; combo group with statin, probucol, and cilostazol	3 years	All three groups showed significant regression of carotid IMT at 3 years compared to baseline. Decrease in mean carotid IMT was significantly greater in the combo group than control group at 1 year
Diabetes mellitus	Sakura (Endo et al., 2013)	Japan	2013	T2DM patients with clinical albuminuria (urinary albumin excretion >300 mg/g creatinine)	162	Probucol treatment (500 mg/d) vs. no probucol treatment	5 years	Scr rate of increase reduced by 0.049 mg/dL/month compared to placebo. Event-free rate was higher in the probucol group (*p* = 0.02)
Diabetes mellitus	Not specified (Zhu et al., 2016)	China	2016	Patients with T2DM and 24-h proteinuria 0.5–3 g	160	Both groups were given telmisartan 80 mg q.d. For 48 weeks. The probucol + telmisartan group was given probucol 500 mg b.i.d. for the first 24 weeks, with the dosage then reduced to 250 mg b.i.d. for the remaining 24 weeks	48 weeks	The 24-h proteinuria levels were significantly reduced in the probucol + telmisartan group compared to the telmisartan group

PCI, percutaneous coronary intervention; IMT, intima-media thickness; EVBT, endovascular brachytherapy; CHD, coronary heart disease; FMD, flow-mediated vasodilation during reactive hyperemia; FH, familial hypercholesterolemia; T2DM, type 2 diabetes mellitus; Scr, serum creatinine.

### 6.2 Forward-facing agenda: addressing research gaps and unlocking future potential

To resolve any lingering questions and determine probucol’s definitive role in modern medicine, a forward-facing research agenda focused on addressing the clear research gaps through innovative clinical trial designs and novel pharmacological strategies is essential. The most critical consideration here is the initiation of a global, large-scale, randomized, placebo-controlled cardiovascular outcomes trial. Such a trial evaluating probucol on top of the current standard of care (including high-intensity statins) is essential to definitively determine if its unique anti-inflammatory and antioxidant properties can significantly reduce residual inflammatory risk and improve the hard clinical endpoints in a high-risk ASCVD population. Ideally, this patient cohort should be enriched for individuals with evidence of high oxidative stress or inflammatory burden (e.g., elevated hs-CRP or Lp-PLA2 levels) to validate the action mechanisms more precisely.

Beyond a definitive outcome trial, future research efforts should move past viewing probucol as a standalone agent and actively explore its synergistic potential in combination therapies. Combining probucol with statins is a logical step in this direction, while partnering it with PCSK9 inhibitors presents a compelling strategy to achieve maximal lipid lowering alongside potent antioxidant effects. Furthermore, combining probucol with new-age anti-inflammatory drugs like colchicine could offer a powerful and dual-pronged approach to target systemic inflammation and lipoprotein oxidation simultaneously. Parallelly, drug-delivery systems present another innovative frontier, particularly the application of nanoformulations. Probucol’s lipophilicity makes it an ideal candidate for nanoencapsulation in targeted nanoparticles (e.g., those designed to home in on inflamed atherosclerotic plaques), which could dramatically enhance its therapeutic index by increasing the local drug concentration at the disease site while minimizing off-target effects like QT prolongation and gastrointestinal intolerance.

In conclusion, probucol is an established drug poised for a renaissance. Although it has a complex past, its unique pharmacological profile aligns perfectly with our modern understanding of ASCVD as a disease entailing both lipid retention and inflammation. By addressing the clear research gaps identified herein through definitive clinical trials and embracing innovative strategies like combination therapy and nanoformulations, the medical community can finally clarify the place of this remarkable molecule in the 21st-century therapeutic arsenal.
