# Supplementary material for: Probucol: revisiting as a multifaceted therapeutic agent in atherosclerosis
Source: Front Pharmacol. 2026 Jan 12;16:1704983. doi: 10.3389/fphar.2025.1704983 (PMC12833333; doi:10.3389/fphar.2025.1704983)
Supplement: Supplementary file 1 [file Supplementaryfile1.docx]

# **4 Probucol in Diabetes Management**

Probucol has a potential to manage diabetes mellitus (DM) complication mainly profit from its polyfunctionality ^[1]^. It actively regulates blood glucose, enhances pancreatic beta cell function, improves insulin sensitivity and endothelial dysfunction ^[2]^ Pr ADDIN EN.CITE . Effect of pr ADDIN EN.CITE ation setting that reduces serum inflammation factors and doubles islets transplants in type 1 DM (T1DM) mouse, suggested enhancing in survival and functions of grafts ^[5]^.

## ***4.1 Probucol in the Management of Diabetic Nephropathy***

Diabetic nephropathy (DN) is influenced by oxidative stress, making probucol a potential treatment. Probucol reverses the overexpression of heat shock protein HO-1 in diabetic rat glomeruli. It also improves redox balance in rodent models of DN ^[6]^. Probucol also prevented AKI in diabetes-induced nephropathy in rats. It could inhibit p66Shc expression via AMPK-SIRT1-AcH3 pathway, ameliorating renal damage caused by DN ^[7]^. Randomized clinical trials showed that combination therapy of probucol with telmisartan has greater benefits than telmisartan monotherapy regarding reduction in urinary protein in DN patients with significant proteinuria throughout their course. Only participants who received combined treatments reduced proteinuria levels at 48 weeks among those whose baseline 24 h-proteinuria was >1.0 g. There were no significant differences between groups concerning adverse CVEs or other events in this study ^[8]^.

## ***4.2 Probucol in the Management of Diabetic Peripheral Neuropathy***

Diabetic peripheral neuropathy (DPN) is a kind of DM complication with nerve damage caused by long-term hyperglycemia. Its pathogenesis involves metabolic disorders, vascular damage, deficiency of neurotrophic factors, oxidative stress, and other factors ^[9]^. Compared to the use of mecobalamin alone, the combination therapy with probucol and mecobalamin can effectively improve patients' scores on the Toronto Clinical Scoring System (TCSS), which includes dimensions of symptoms, sensation, and reflexes ^[10]^. This combined treatment can improve nerve conduction velocity, a key indicator of nerve function status, and can positively affect oxidative stress markers, which are particularly important in the management of DPN.

## ***4.3 Probucol in the Management of Diabetic Retinopathy***

Probucol's role in diabetic retinopathy management is highlighted by its ability to preserve the function of the large-conductance calcium-activated potassium channel (BKCa) in DM rat models, which may prevent or slow retinal circulation damage ^[11]^. It also slows hyperglycemia-induced cataract progression, mainly by reducing oxidative damage rather than through lipid-lowering effects. The protective effect of probucol may also involve activating the Nrf2/p62 signaling pathway and upregulating the anti-apoptotic protein Bcl2, thereby protecting Müller cells from damage induced by a high-sugar environment ^[12]^.

# **5 Probucol's Therapeutic Potential in Non-alcoholic Fatty Liver Disease**

Non-alcoholic fatty liver disease (NAFLD) also involves numerous risk factors including mitochondrial dysfunction and oxidative stress ^[13]^. In monosodium glutamate-induced fatty liver animal models, probucol significantly reduced liver fat levels, and showed protective effects on carbon tetrachloride-induced liver injury ^[68]^. Studies explored probucol's effect in rat NASH models after high-fat diet for nonalcoholic steatohepatitis development, their results showed the benefit of probucol to mitigate NASH through enhancing insulin sensitivity and decreasing oxidative stress and inflammation ^[69,70]^. In carbon tetrachloride (CCl4)-induced liver fibrosis mouse models, probucol decreased fibrosis and autophagy while upregulating the farnesoid X receptor and inhibiting hepatic stellate cells (HSC) activation in vitro, indicating its potential to alleviate liver fibrosis through the inhibition of autophagy and HSC activity ^[14]^. Clinical studies have confirmed probucol's efficacy in NASH where it lowered aminotransferase and normalized grading of a fatty liver after 1-year therapy. Furthermore, when combining probucol and pantethine, it was proven safe and efficacious in ameliorating liver inflammation and fibrosis ^[72]^. An open-label preliminary study confirmed that probucol improves liver function by alleviating insulin resistance and oxidative stress ^[73]^. Due to current NAFLD treatments not being ideal there has been an increasing interest in further research on probucol, we expect more exploration would confirm probucol as a promising therapeutic option for patients suffering from NAFLD ^[74]^.

# **6 Probucol in Other Diseases**

With scientists' increasing knowledge about probucol, more and more people began to utilize it in various disease treatment studies. More special application field for probucol has been explored one step after another now: such as neurodegenerative diseases^[75]^, kidney injury^[76]^, cancer ^[77]^, inflammation ^[78]^, osteoporosis ^[79]^, malaria ^[80]^, lung injury ^[81]^, even the cure of infertility ^[82]^. Although these studies are primarily focused on preclinic, they still indicate the broad applications of probucol. This article mainly introduces the potential therapeutic effects of probucol in neurodegenerative diseases, renal injury, and its antitumor actions.

## ***6.1 Probucol in Neurodegenerative Disease Management***

Probucol’s neuro-protective effects are due to synergistic antioxidant, anti-inflammatory and BBB-stabilizing effects that maintain the function of neurons and cognition^[75]^. It reduces injury caused by oxidative stress through Nrf2/Keap1 and ROS-scavenging, inhibits neuro-inflammation and levels of apoptosis marker simultaneously^[83]^. It improves cerebral hemodynamics and neuropathological burden in AD, PD and HD animals. Moreover, in AD model, probucol regulates Aβ lipoprotein metabolism, upregulates peripheral clearance mechanism which may delay amyloid accumulation. The ongoing PIA Phase II trial will evaluate its cognitive protection and effect on Amyloid Load ^[84]^. It protects dopaminergic neuron from neurotoxin induced damages in PD animal models which is reflected through maintenance of Tyrosine hydroxylase, Glutathione system ^[85]^. For HD application, it showed oxidative stress attenuation response through GPx activation^[86]^. Increasing evidences indicate new applications in metabolism associated neurodegeneration ^[87]^, recent mechanistic investigations revealed the importance of ABCA1 mediated lipid trafficking and mitochondrial-lipid droplet interconnection in probucol's neuro-protective effect^[88]^.

## ***6.2 Probucol's Renal Protective Effects***

Probucol not only exhibit renoprotective effects on DN, but also on the other CKD models. Its experimental results demonstrated that it could lower proteinuria and protect glomeruli from injury through ways related to the pharmacological mechanisms as described above ^[89]^. A multicenter study has reported 27% partial remission rate on resistant patients with membranous nephropathy after treated with probucol. Furthermore, using valsartan alone was inferior to combination therapy in lowering proteinuria in IgA nephropathy ^[76]^. This effect is also manifested in non-diabetic CKD patients taking probucol together with RAS inhibitors which improved their insulin sensitivity ^[90]^.

This drug will likely prove helpful at dealing with both nephrotic hyperlipidemia and CKD progression. These two kinds of modifications can jointly prevent occurrence of pathological damage to vessels, reinforcing positive therapeutic effects to develop atherosclerosis. These collective actions position probucol as a promising multi-target agent for cardiorenal protection, warranting further investigation into its optimal clinical application and combination strategies.

## ***6.3 Probucol's Anti-cancer Potential***

Cancer's a tricky foe but often fueled by chronic inflammation and oxidative stress. But probucol can put the brakes on cancer cell proliferation. It got a special knack for ovarian cancer cells by meddling with their cell cycle and tamping down the NF-κB and MAPK signaling pathways^[15]^. It is kinder in general toward other types of cancer lines but can be more of a thorn toward Multiple Myeloma cells - specifically H929 and RPMI8226, though it might have an improvement ^[91]^. Enter the "Directed Nanoassembly of Probucol " system-a smart move that ups probucol's oral bioavailability and could change the game for treating breast cancer that's spread to the lungs ^[16]^.

[1] An Y, Xu BT, Wan SR, et al. The role of oxidative stress in diabetes mellitus-induced vascular endothelial dysfunction. Cardiovasc Diabetol. 2023;22: 237. doi:10.1186/s12933-023-01965-7

[2] Gorogawa S, Kajimoto Y, Umayahara Y, et al. Probucol preserves pancreatic beta-cell function through reduction of oxidative stress in type 2 diabetes. Diabetes Res Clin Pract. 2002;57: 1-10. doi:10.1016/s0168-8227(02)00005-0

[3] Mooranian A, Zamani N, Takechi R, et al. Probucol-poly(meth)acrylate-bile acid nanoparticles increase IL-10, and primary bile acids in prediabetic mice. Ther Deliv. 2019;10: 563-571. doi:10.4155/tde-2019-0052

[4] Guttapadu R, Korla K, Uk S, Annam V, Ashok P, Chandra N. Identification of Probucol as a candidate for combination therapy with Metformin for Type 2 diabetes. NPJ Syst Biol Appl. 2023;9: 18. doi:10.1038/s41540-023-00275-8

[5] Mooranian A, Ionescu CM, Wagle SR, et al. Probucol Pharmacological and Bio-Nanotechnological Effects on Surgically Transplanted Graft Due to Powerful Anti-Inflammatory, Anti-Fibrotic and Potential Bile Acid Modulatory Actions. Pharmaceutics. 2021;13, doi:10.3390/pharmaceutics13081304

[6] Ma X, Jiao Z, Liu Y, et al. Probucol Protects Against Contrast-Induced Acute Kidney Injury via the Extracellular Signal-Regulated Kinases 1 and 2 (ERK1/2)/JNK-Caspase 3 Pathway in Diabetic Rats. Med Sci Monit. 2019;25: 1038-1045. doi:10.12659/msm.913106

[7] Yang S, Zhao L, Han Y, et al. Probucol ameliorates renal injury in diabetic nephropathy by inhibiting the expression of the redox enzyme p66Shc. Redox Biol. 2017;13: 482-497. doi:10.1016/j.redox.2017.07.002

[8] Zhu H, Chen X, Cai G, et al. Telmisartan combined with probucol effectively reduces urinary protein in patients with type 2 diabetes: A randomized double-blind placebo-controlled multicenter clinical study. J Diabetes. 2016;8: 677-85. doi:10.1111/1753-0407.12347

[9] Selvarajah D, Kar D, Khunti K, et al. Diabetic peripheral neuropathy: advances in diagnosis and strategies for screening and early intervention. Lancet Diabetes Endocrinol. 2019;7: 938-948. doi:10.1016/s2213-8587(19)30081-6

[10] Peng HY, Gong YY. Analysis of the effect of probucol-mecobalamin tablets combination on oxidative stress in patients with diabetic peripheral neuropathy. Neurosci Lett. 2021;741: 135484. doi:10.1016/j.neulet.2020.135484

[11] Liu HW, Luo Y, Zhou YF, Chen ZP. Probucol Prevents Diabetes-Induced Retinal Neuronal Degeneration through Upregulating Nrf2. Biomed Res Int. 2020;2020: 3862509. doi:10.1155/2020/3862509

[12] Zhou YF, Liu HW, Yang X, Li CX, Chen JS, Chen ZP. Probucol attenuates high glucose-induced Müller cell damage through enhancing the Nrf2/p62 signaling pathway. Int Ophthalmol. 2023;43: 4595-4604. doi:10.1007/s10792-023-02859-z

[13] Pafili K, Roden M. Nonalcoholic fatty liver disease (NAFLD) from pathogenesis to treatment concepts in humans. Mol Metab. 2021;50: 101122. doi:10.1016/j.molmet.2020.101122

[14] Yang R, Hu Z, Zhang P, et al. Probucol ameliorates hepatic stellate cell activation and autophagy is associated with farnesoid X receptor. J Pharmacol Sci. 2019;139: 120-128. doi:10.1016/j.jphs.2018.12.005

[15] Chuang LY, Guh JY, Ye YL, Lee YH, Huang JS. Effects of probucol on cell proliferation in human ovarian cancer cells. Toxicol Res (Camb). 2016;5: 331-339. doi:10.1039/c5tx00088b

[16] Zhang Z, Cao H, Jiang S, et al. Nanoassembly of probucol enables novel therapeutic efficacy in the suppression of lung metastasis of breast cancer. Small. 2014;10: 4735-45. doi:10.1002/smll.201400799
