# Supplementary material for: Probucol: revisiting as a multifaceted therapeutic agent in atherosclerosis
Source: Front Pharmacol. 2026 Jan 12;16:1704983. doi: 10.3389/fphar.2025.1704983 (PMC12833333; doi:10.3389/fphar.2025.1704983)
Supplement: Supplementary file 2 [file Table1.docx]

**Supplementary Materials Table 1.** A Comparative Summary of Probucol's Effects in Preclinical versus Clinical Studies

| Domain of Action | Preclinical Evidence (Mechanistic Insights from In Vitro & In Vivo Models) | Clinical Evidence (Observed Effects in Human Studies) |
| --- | --- | --- |
| Lipid Profile Modulation | LDL-C: Moderately reduces plasma LDL cholesterol by promoting its catabolism through LDL receptor-independent pathways.  HDL-C: Markedly reduces HDL cholesterol by inhibiting the synthesis of apolipoproteins A-I and A-II and accelerating HDL catabolism. | LDL-C: Confirmed moderate reduction (typically 10-15%).  HDL-C: Confirmed significant reduction (typically 20-30%), which has been a major point of clinical concern and controversy.  Triglycerides: Variable and generally modest effects. |
| Antioxidant Activity | Direct Effect: Acts as a potent, lipophilic, chain-breaking antioxidant that integrates into lipoprotein particles.  Mechanism: Directly inhibits the copper-mediated oxidation of LDL, preventing the formation of pro-atherogenic oxidized LDL (ox-LDL).  Tissue Effect: Reduces markers of oxidative stress (e.g., MDA) in aortic tissue and plasma. | Confirmed Mechanism: Greatly increases the resistance of patients' LDL to ex vivo oxidation.  Biomarker Reduction: Significantly lowers circulating levels of ox-LDL and autoantibodies against ox-LDL, confirming systemic antioxidant action in humans. |
| Anti-inflammatory Effects | Cellular Level: Downregulates the expression of key vascular adhesion molecules (VCAM-1, ICAM-1) on endothelial cells.  Molecular Level: Inhibits monocyte chemotaxis (↓MCP-1) and suppresses inflammatory signaling pathways (e.g., NF-κB).  In Vivo: Reduces macrophage infiltration and inflammatory cytokine expression within atherosclerotic plaques. | Biomarker Reduction: Shown to decrease levels of systemic inflammatory markers such as C-reactive protein (CRP), serum amyloid A (SAA), and interleukin-6 (IL-6).  Functional Effect: Evidence suggests improved endothelial-dependent vasodilation, a functional measure of reduced vascular inflammation. |
| Direct Anti-Atherosclerotic Effects | Primary Finding: Dramatically reduces atherosclerotic lesion formation (by up to 80-90%) in multiple animal models (rabbits, primates, apoE-/- mice), often independent of the degree of plasma cholesterol reduction.  Cellular Effect: Strongly inhibits macrophage foam cell formation. | Surrogate Endpoints: Landmark trials (esp. in Japan) have demonstrated regression of carotid intima-media thickness (CIMT) and regression of Achilles tendon xanthomas.  Post-PCI: Significantly reduces in-stent restenosis rates after coronary angioplasty, confirming a direct vasculoprotective effect. |
| Plaque Stability | Compositional Changes: Promotes a more stable plaque phenotype: increases fibrous cap thickness, boosts collagen content, and reduces the size of the lipid-rich necrotic core.  Cellular Content: Decreases macrophage and foam cell content within established plaques. | Largely Indirect Evidence: While direct histological proof in humans is scarce, the reduction in restenosis and regression of CIMT indirectly support a plaque-stabilizing effect.  Findings from intravascular ultrasound (IVUS) studies have been suggestive but not definitive. |
| Major Adverse Cardiovascular Events (MACE) | Not Applicable: Animal models are typically not designed or powered to measure clinical endpoints like myocardial infarction or stroke. | The Core Controversy: Major Western cardiovascular outcome trials (CVOTs) failed to demonstrate a statistically significant reduction in MACE.  This discrepancy between potent effects on surrogate markers and the lack of benefit on "hard" clinical endpoints led to its withdrawal from many markets. |
